# *Primulina papillosa* (Gesneriaceae), a new species from limestone areas of Guangxi, China

**DOI:** 10.3897/phytokeys.177.63878

**Published:** 2021-05-12

**Authors:** Zi-Bing Xin, Wei-Chuen Chou, Stephen Maciejewski, Long-Fei Fu, Fang Wen

**Affiliations:** 1 Guangxi Key Laboratory of Plant Conservation and Restoration Ecology in Karst Terrain, Guangxi Institute of Botany, Guangxi Zhuang Autonomous Region and Chinese Academy of Sciences, CN-541006, Guilin, China Guangxi Institute of Botany, Chinese Academy of Sciences Guilin China; 2 National Gesneriaceae Germplasm Resources Bank, Guangxi Institute of Botany, Guangxi Zhuang Autonomous Region and Chinese Academy of Sciences, CN-541006, Guilin, China Gesneriad Conservation Center of China Guilin China; 3 Gesneriad Committee, China Wild Plant Conservation Association, CN-541006, Guilin, China The Gesneriad Society Philadelphia United States of America; 4 Gesneriad Conservation Center of China (GCCC), Guilin Botanical Garden, Guangxi Zhuang Autonomous Region and Chinese Academy of Sciences, CN-541006, Guilin, China Guangxi Key Laboratory of Plant Conservation and Restoration Ecology in Karst Terrain, Guangxi Institute of Botany, Guangxi Zhuang Autonomous Region and Chinese Academy of Sciences Guilin China; 5 The Gesneriad Society, 2030 Fitzwater Street, Philadelphia, PA. 19146-1333, USA Guangxi Institute of Botany, CAS Guilin China

**Keywords:** Cliff-dwelling, flora of Guangxi, limestone flora, new taxon, taxonomy

## Abstract

*Primulina papillosa* Z.B. Xin, W.C. Chou & F. Wen, a new species from limestone areas of Guangxi, China, is described and illustrated here. It morphologically resembles *P. linearifolia* (W.T. Wang) Yin Z. Wang and *P. pseudolinearifolia* W.B. Xu & K.F. Chung, but can be easily distinguished by some combined characters, especially its leaf blades densely papillose-hispid. We found only one population at the type locality with no more than 200 individuals, so that this new species is provisionally assessed as Critically Endangered (CR) using IUCN Criteria.

## Introduction

By the end of 2020, the genus *Primulina*Hance (1883) of the family Gesneriaceae comprised 201 species and 27 varieties (IPNI 2021; Tropicos 2021). It is mainly distributed throughout southern, south-western China and northern Vietnam (Wang et al. 2011; Weber et al. 2011). China is the centre of diversity for *Primulina* with at least 183 species and 27 varieties occurring there at present, especially in limestone areas (e.g. Wei 2018; Wen et al. 2019, 2021; Ge et al. 2020; Liu et al. 2020; Xin et al. 2020a, b, c). The tropical and subtropical karst limestone mountainous areas of Guangxi are the centres of species diversity and differentiation of this genus (Li et al. 2019). An acceleration of *Primulina* species discovery has been seen over the last five years, with an average of over ten new species per year (Wen et al. 2019, 2021). Assuming this trend persists, more new *Primulina* species from China will most likely be discovered (Möller 2019).

A Gesneriaceae enthusiast from Guangxi found this unknown plant species in the wild on 6 April 2020. One of authors, W.C. Chou, went to the type locality and collected the specimens for it. At the same time, some living plants were introduced and cultivated in the gardens of the Gesneriad Conservation Center of China (GCCC) and National Gesneriaceae Germplasm Resources Bank for further study. Detailed comparisons of the specimens and living plant materials with the type specimens and protologues of some related known *Primulina* species revealed that these specimens neither fit the existing protologues nor conform to the type specimens of these species. Nevertheless, its leaf shape and rhizome are most similar to those of *P. linearifolia* (W.T. Wang) Yin Z. Wang (Wang and Pan 1982; Wang et al. 2011) and *P. pseudolinearifolia* W.B. Xu & K.F. Chung (Xu et al. 2011, 2012) and it can be easily distinguished from the latter two by the combination of several morphological characters (Table 1), especially its leaf blades densely papillose-hispid. Thus, we confirmed that it represents a new species of *Primulina* and describe it here.

**Table 1.** Detailed comparison of *Primulina papillosa* and its two relatives.

Characters	*P. papillosa*	*P. linearifolia*	*P. pseudolinearifolia*
Leaf blades	densely papillose-hispid	densely appressed pubescent	densely appressed pubescent
Cymes	1–2-flowered	4–7-flowered	4–12-flowered
Pedicel	20–35 mm long	5–12 mm long	7–15 mm long
Calyx lobes	7.5–9 × ca. 2 mm	3.2–4 × 0.6–1.1 mm,	5–6 × ca. 1 mm
Central staminodes	ca. 0.5 mm long	none	ca. 3 mm long
Disc	ca. 1.2 mm high, margin entire	ca. 0.5 mm high, margin repand	ca. 2.5 mm high, margin repand
Flowering time	September to November	April	April to May
Capsule	5–6.5 cm long	2.2–3.6 cm long	3–4.5 cm long

## Taxonomic treatment

### Primulina papillosa

Taxon classificationPlantaeLamialesGesneriaceae

Z.B. Xin, W.C. Chou & F. Wen sp. nov.

B3817975-4E85-5924-A5FE-780D9887F1AF

urn:lsid:ipni.org:names:77217100-1

Figs 1 , 2E–F

#### Diagnosis.

The new species resembles *Primulina linearifolia* (Fig. 2A, B) and *P. pseudolinearifolia* (Fig. 2C, D), but can be easily distinguished from the latter two by both surfaces of its leaf blades being densely papillose-hispid. It differs from *P. linearifolia* by its 1–2-flowered per cyme (vs. 4–7-flowered); pedicel 20–35 mm long (vs. 5–12 mm); calyx lobes 7.5–9 mm long (vs. 3.2–4 mm); disc ca. 1.2 mm high, margin entire (vs. ca. 0.5 mm, margin repand); capsule 5–6.5 cm long (vs. 2.2–3.6 cm). It also differs from *P. pseudolinearifolia* by its 1–2-flowered per cyme (vs. 4–12-flowered); pedicel 20–35 mm long (vs. 7–15 mm); central staminodes ca. 0.5 mm long (vs. ca. 3 mm); disc ca. 1.2 mm high, margin entire (vs. ca. 2.5 mm, margin repand).

**Figure 1.**
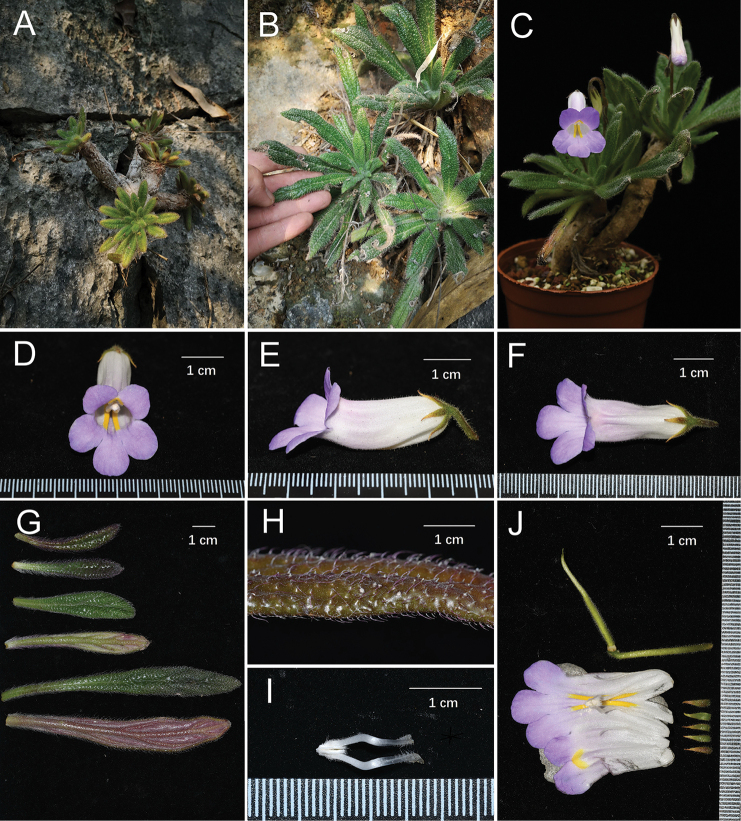
*Primulina papillosa***A, B** habitat **C** habit **D** front view of the corolla **E** lateral view of the corolla **F** top view of the corolla **G** adaxial and abaxial surface of leaf blades **H** papillose-hispid hairs on leaf blade surface **I** stamens **J** pistil, calyx and opened corolla with stamens and staminodes. (**A, B** photos by W.C. Chou, **C–J** photos by F. Wen; arranged by Z.B. Xin).

#### Type.

China. Guangxi: cultivated material in the Gesneriad Conservation Center of China and National Gesneriaceae Germplasm Resources Bank, harvested on 24 October 2020, wild-collected, from Dingdang Town, Longan County, Nanning City, 23°07'N, 107°57'E, 9 April 2020, *W.C. Chou 20200409-01* (Holotype, IBK!; Isotypes, IBK!).

**Figure 2.**
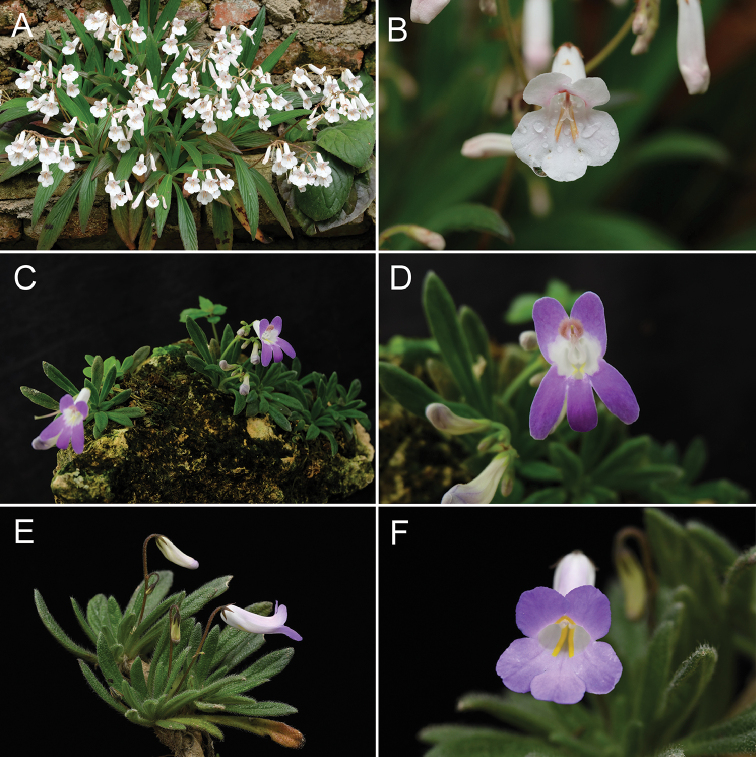
Comparison of three species of *Primulina***A, B***P. linearifolia***C, D***P. pseudolinearifolia***E, F***P. papillosa*. (photos by F. Wen; arranged by Z.B. Xin).

#### Description.

Herbs perennial. ***Rhizome*** thickened, woody, subterete, 10–15 cm long, 1.5–2 cm in diameter, internodes inconspicuous, commonly branched at the apex of the rhizome or not branched. ***Leaves*** 15–25, congested at the apex of the rhizome, subsessile; ***leaf blade*** fleshy, linear-lanceolate, 5–15 × 0.9–1.8 cm, densely papillose-hispid on both surfaces, apex obtuse to round, base attenuate, margin entire, lateral veins 2–4 on each side of the mid-rib, conspicuous on the abaxial surface, inconspicuous on the adaxial surface. ***Cymes*** 2–5, axillary, 1–2-flowered; peduncle 4–8 cm long, ca. 2 mm in diameter, glandular-pubescent and sparsely pilose; ***bracts*** 2, opposite, linear-lanceolate, 6–8 × 1–1.5 mm, apex acute, margin entire, pubescent on both surfaces, pedicel 2–3.5 cm long, ca. 2 mm in diameter, glandular-pubescent. ***Calyx*** 5-parted from the base, segments equal, lanceolate, 7.5–9 × ca. 2 mm, abaxially glandular-pubescent, adaxially sparsely glandular-pubescent to glabrous, apex acute, margin entire. ***Corolla*** purple, throat with two yellow stripes inside, 3.5–4.5 cm long, outside puberulent with both glandular and eglandular hairs, inside glabrous, tube 2.5–3 cm long, orifice 0.8–1.5 cm in diameter; limb distinctly 2-lipped, adaxial lip 2-parted to the middle, with a yellow patch between the two adaxial lobes, lobes ovate, 6–7 × 8–9 mm, abaxial lip 3-parted to near the base, lobes ovate, 8–9 × 9–10 mm. ***Stamens*** 2, adnate ca. 1.2 cm above the corolla base; filaments 1.3–1.5 cm long, geniculate near the middle, sparsely pubescent; anthers reniform, 3.5–4 mm long, bearded; ***staminodes*** 3, lateral ones linear, glabrous, ca. 9 mm long, apex capitate, sparsely pubescent, adnate to ca. 1 cm above the corolla tube base, the central one ca. 0.5 mm long, apex capitate, adnate to 3.5 mm above the corolla tube base. ***Disc*** annular, ca. 1.2 mm high, margin entire, glabrous. ***Pistil*** 2.5–3 cm long, ***ovary*** 1.4–1.6 cm long, ca. 2 mm in diameter, densely glandular-pubescent and eglandular-pubescent; ***style*** 0.9–1.2 cm long, 1.5 mm in diameter, glandular-pubescent and eglandular-pubescent; ***stigma*** obtrapeziform, ca. 2 mm long, apex shallowly 2-lobed. ***Capsule*** linear, 5–6.5 cm long, 2–3 mm in diameter, puberulent with both glandular and eglandular hairs.

#### Phenology.

Flowering from September to November, fruiting from October to December.

#### Etymology.

The specific epithet ‘*papillosa*’ is derived from the leaf blade densely papillose-hispid on both surfaces.

#### Vernacular name.

The Chinese name ‘刺疣报春苣苔’ (Cì Yóu Bào Chūn Jù Tái) is newly coined for this species because of its special leaf blades surface full of densely papillose-hispid hairs.

#### Distribution and habitat.

*Primulina papillosa* is only known from the type locality, Dingdang Town, Longan County, Nanning City, Guangxi, China. It only grows in crevices of the cliff near the top of limestone hills in a subtropical evergreen seasonal rain forest.

#### Conservation status.

*Primulina papillosa* is only found from the type population with less than 200 individuals. The EOO and AOO of the new species are about 1.05 km^2^ and 0.01 km^2^, respectively. The beautiful flowers, thickened rhizomatous woody stem and leaves with dense papillose-hispid hairs, have led to its over-harvesting by local people who have sold it as an ornamental plant. Furthermore, the natural habitat is mostly disturbed due to local farmers imposing intense pressure on the remaining patches of primary forest. Thus, following the IUCN Red List Categories and Criteria (IUCN 2019), it is temporarily assessed as Critically Endangered [CR B1+B2ab (iii, v)].

## Supplementary Material

XML Treatment for Primulina papillosa
